# Noninvasive measures of physiological stress are confounded by exposure

**DOI:** 10.1038/s41598-019-55715-5

**Published:** 2019-12-16

**Authors:** Diana J. R. Lafferty, Marketa Zimova, Lindsay Clontz, Klaus Hackländer, L. Scott Mills

**Affiliations:** 1 Wildlife Ecology and Conservation Science Lab, Department of Biology, Northern Michigan University, Marquette, MI 49855 USA; 2 Fisheries, Wildlife, and Conservation Biology Program, Department of Forestry and Environmental Resources, North Carolina State University, Raleigh, NC 27695 USA; 3 Wildlife Biology Program, University of Montana, Missoula, MT 59812 USA; 4 School for Environment and Sustainability, Institute for Global Change Biology, University of Michigan, Ann Arbor, MI 48104 USA; 5 Institute of Wildlife Biology and Game Management, University of Natural Resources and Life Sciences, Vienna, Gregor-Mendel-Str. 33, 1180 Vienna, Austria; 6 Office of Research and Creative Scholarship, University of Montana, Missoula, MT 59812 USA

**Keywords:** Ecophysiology, Animal physiology

## Abstract

Glucocorticoids and glucocorticoid metabolites are increasingly used to index physiological stress in wildlife. Although feces is often abundant and can be collected noninvasively, exposure to biotic and abiotic elements may influence fecal glucocorticoid metabolite (FGM) concentrations, leading to inaccurate conclusions regarding wildlife physiological stress. Using captive snowshoe hares (*Lepus americanus*) and simulated environmental conditions, we evaluated how different realistic field conditions and temporal sampling constraints might influence FGM concentrations using an 11-oxoetiocholanolone-enzyme immunoassay. We quantified how fecal pellet age (i.e., 0–6 days), variable summer temperatures, and precipitation affected FGM concentrations. Fecal pellet age had a strong effect on FGM concentrations (β_Age_ = 0.395, s.d. = 0.085; β^2^_Age_ = −0.061, s.d. = 0.012), which were lowest at the beginning and end of our exposure period (e.g., mean_day6_ = 37.7 ng/mg) and typically highest in the middle (mean_day3_ = 51.8 ng/mg). The effect of fecal pellet age on FGM concentrations varied across treatments with warm-dry and cool-wet conditions resulting in more variable FGM concentrations relative to control samples. Given the confounding effects of exposure and environmental conditions, if fresh fecal pellet collection is not an option, we encourage researchers to develop a temporally consistent sampling protocol to ensure all samples are exposed to similar environmental conditions.

## Introduction

Understanding wildlife responses to local and broad-scale environmental perturbations poses an enormous challenge for scientists, wildlife managers, and policy makers. The current need for reliable tools that can be used to measure and monitor wildlife responses to environmental stressors is exacerbated further by ongoing global environmental change. Over the past decade, assessments of adrenocortical activity, particularly measures of glucocorticoids (stress hormones) and glucocorticoid metabolites, have been used increasingly to index the stress burden experienced by diverse taxa^1–6^. In fact, fecal glucocorticoid metabolites (FGM) have been shown to provide an integrative measure of circulating glucocorticoids, representing the physiological state of the target animal over a well-defined period of time^7^. Feces is a particularly useful matrix for measuring stress hormone metabolite concentrations because it is relatively abundant and can be collected with minimal disturbance to study animals^1,5,7^, thus eliminating the confounding effects of capture stress^8–10^.

While measures of FGM concentrations have great potential to contribute to integrative wildlife conservation and management^11^, important sampling caveats that can affect stress levels estimated from fecal samples must be considered^1,4,5,12^. Whether an increase, decrease or no changes in FGM concentration is detected within fecal samples from a target population often depends on multiple factors including: 1) the species and sex of the individuals being sampled; 2) the species-specific and sex-specific glucocorticoid metabolism in combination with the immunoreactivity of the antibody utilized in the enzyme immunoassay (EIA); 3) the steroid extraction method applied to measure FGM concentrations^7^. For example, variation in measures of FGM concentrations may result from differences in immunoreactivity of the numerous glucocorticoid metabolites present in feces, particularly if feces has been exposed to water^7,13,14^. Because water is highly polar, samples exposed to precipitation may result in more rapid extraction of the most polar metabolites, followed later by less polar metabolites^13^. As such, the effects of water exposure on FGM concentrations will in turn depend on the affinities of the antibody used to measure the FGMs in the sample^7,13,14^. However, measures of FGM concentrations may also be influenced by environmental factors. For instance, ambient temperature and humidity can affect bacterial metabolization that can result in possible increases or decreases in FGM concentrations^14–16^. Differences in microclimates across the range of American pika (*Ochotona princeps*) have been reported to influence FGM concentrations when feces are not immediately collected and preserved after defecation^17^. Hot and dry environmental conditions were associated with decreased FGM concentrations in cheetah (*Acinonyx jubatus*)^14^, whereas precipitation seemed to cause a “washing-out effect” in which FGM leached from the feces of mountain hare (*Lepus timidus*)^18^. However, a recent study that evaluated the suitability of different EIAs for monitoring adrenocortical activity in leopards (*Panthera pardus*), found that FGM concentrations remained relatively stable over a six-day period post defecation^19^. Evidently, the confounding effects of exposure time and abiotic environmental conditions are variable by species and in some situations mitigated by observing animals and collecting samples immediately after defecation^20,21^ or possibly by sampling feces deposited in snow^22^. However, observing defecation may be logistically challenging for a variety of taxa, and the collection of feces on snow is not possible impossible outside of the snow season or in the tropics. Thus, understanding how environmental conditions affect FGM concentrations is both complex and important for developing rigorous noninvasive sampling protocols for any wildlife study.

We use snowshoe hares (*Lepus americanus*), as an example to illustrate the variable influence of time and environmental conditions on FGM concentrations. Snowshoe hares are particularly suitable for our study for three main reasons. First, from an eco-evolutionary perspective, snowshoe hares have emerged recently as a model organism for understanding the effects of climate change on seasonal coat color changing species^23–26^. However, before scientists can evaluate whether snowshoe hares exhibit a biologically meaningful stress response to ongoing climate change, we must first establish rigorous sampling protocols for obtaining representative samples from which measures of physiological stress can be indexed. Second, a protocol for quantifying FGM concentrations from fecal material using an 11-oxoetiocholanolone-EIA is well established^27^ and validated for snowshoe hares^28^. Third, results from previous research determined the lag time between stress-induced cortisol production and the expression of cortisol metabolites in snowshoe hare fecal pellets is approximately eight hours, thus providing a sampling frame over which an animal may be held in a trap during field-based operations^28^, thereby allowing us to conduct a controlled experiment that closely mimics potential field constraints.

Using a captive collection of snowshoe hares and simulated environmental conditions, we examined the effect of realistic field conditions and temporal sampling constraints on FGM measurements. First, we tested how fecal pellet age (i.e., exposure time of 0–6 days), temperature (summer warm [23.5 °C], summer cool [7.3 °C]) and precipitation (dry, wet) affected FGM concentrations. Second, we evaluated at what fecal pellet age FGM levels begin to differ from FGM concentrations in control samples when exposed to different environmental treatments (precipitation and temperature combinations). Finally, we provide recommendations for field study design to ensure measures of FGM concentrations represent the physiological state of the target animal and/or populations.

## Results

We found that the age of feces affects FGM concentrations, and the age-FGM relationship has a curvilinear shape (β_Age_ = 0.395, s.d. = 0.085; β^2^_Age_ = −0.061, s.d. = 0.012). FGM concentrations peaked on day 3 (mean_day3_ = 51.8 ng/mg, s.d. = 24.8) and were lowest on day 6 (mean_day6_ = 37.7 ng/mg, s.d. = 24.6). Temperature, precipitation and sex had no significant effect on FGM concentrations during those days (alpha set at 0.05).

**Figure 1:**
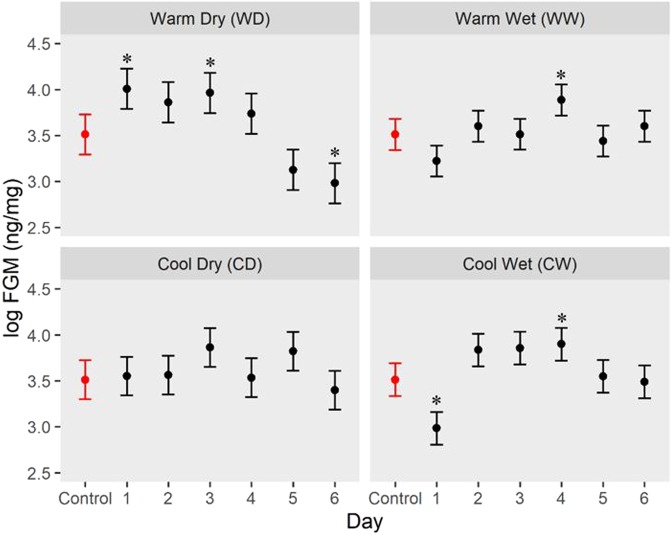
Modeled snowshoe hare FGM concentrations (ng/mg; log-transformed) over time subject to different environmental treatments. Points show means and bars show standard errors across all individuals each day (*n* = 15). Control FGM concentrations, shown in red, were quantified from samples collected within eight hours of defecation and are the same in each panel. Modeled FGM concentrations are based on measured FGM concentrations from pellets exposed to different environmental conditions, shown in black. Statistically significant differences between the exposed samples and the controls are indicated by the asterisks (alpha level = 0.05). Figure created by S. J. Gillman.

We found multiple significant differences between the FGM concentrations in the control samples and FGM concentrations exposed to the environmental treatments (Fig. 1). We did not detect a particular age at which FGM concentrations consistently began to differ from the control samples (i.e., day 0). Instead, we found substantial variation in the trend of FGM concentration over time across all experimental treatments. For example, in the CW treatment, FGM concentrations initially declined and then followed the curvilinear trajectory, whereas FGM concentrations in the CD treatment were the most consistent over the six-day exposure period. Overall, we found that FGM concentrations differed from those measured in the control samples most often in the WD (on three days) and the CW (on two days) (Fig. 1).

## Discussion

Our results indicate that the length of time between snowshoe hare defecation (i.e., pellet age) and fecal sample collection can affect FGM measurements. For example, our data from the WD treatment show an initial increase in FGM concentrations over the first four days followed by a decline in FGM concentrations over the last two days (Fig. 1). However, both WW and CW treatments resulted in an initial decline in measures of FGM concentrations before rising to concentrations similar to or higher than FGM concentrations measured from control samples (Fig. 1), which likely resulted from fecal pellet exposure to simulated precipitation and subsequent effects on immunoreactivity associated with polarity. For instance, water is highly polar and thus subjecting samples to simulated precipitation might result the rapid elution of the most polar metabolites, followed by the less polar metabolites^13^. While the effects of precipitation exposure on measures of FGM concentrations may dissipate over time, thus resulting in FGM concentrations returning to near-baseline levels, the period of time over which this occurs is somewhat variable and must be considered when collecting samples representing various ages, particularly if samples were exposed to different levels of moisture over different periods of time. The observed changes in FGM concentrations under different temperature and precipitation treatments illuminate the importance of obtaining fresh fecal samples or samples collected within the same time frame whenever possible.

Results from our controlled experiment also demonstrate that the effect of fecal pellet age varies across different environmental conditions. For example, the effect of fecal pellet age was strongest under warm dry and cool wet conditions, where the measured FGM differed most frequently from the control samples (Fig. 1). On the other hand, FGM concentrations were least variable over the six-day exposure period represented by a cool and dry summer conditions (Fig. 1). Results from earlier studies of diverse taxa have shown that increased temperatures sometimes result in higher FGM concentrations relative to control samples^14,17,29^. On the other hand, the effects of precipitation (or moisture) on FGM concentrations are variable, leading to increased or decreased FGM concentrations^15,17,30^. Both of our experimental temperature treatments that included simulated precipitation resulted in initial FGM decreases, which may have been the result of a ‘washing-out’ effect in which our application of precipitation removed some FGMs from the sample or possibly a consequence of issues associated with metabolite polarity^7,13,14^. Furthermore, the effects of temperature and precipitation on the FGM concentrations may interact; for example, increased microbial activity in warmer temperatures may be offset by FGM degradation or potential washout when feces are exposed to precipitation. However, whether measures of FGM concentrations show an increase, decrease or no change, is complex and may be related to species, sex, or species-sex specific glucocorticoid metabolism and even to the specific extraction method^7^. In conclusion, it is critical to reduce sample exposure to uncontrolled environmental conditions that may influence measurements of FGM concentrations.

Given the confounding effect of pellet exposure time on FGM measurements, our study demonstrates the need to preserve snowshoe hare feces immediately following defecation. Whenever collection of fresh feces is not possible, as often the case with free-ranging wildlife, including snowshoe hares, we strongly advise researchers apply a systematic sampling protocol to ensure all sampled feces are subjected to similar exposure times and environmental conditions prior to sample preservation. For example, if environmental conditions are stable, line transects or plots could be cleared of feces initially and re-checked for fecal pellet samples within a specified timeframe (e.g., one day, two days). Landscape cloths deployed for short time periods may also be an efficient way to collect feces of known age^31^. For snowshoe hares specifically, we recommend sampling feces during cool dry conditions in the summer. More generally, we advise against opportunistic use of samples collected for purposes other than for measuring FGM concentrations unless the collected samples are of known age (e.g., one day, two days) and collected under similar environmental conditions throughout the sampling period. Samples of unknown age or collected during variable environmental conditions may not accurately reflect the physiological state of the animals sampled and should not be used to infer stress responses or should be used very cautiously when drawing conclusions about the stress response of the target population. Noninvasive measures of physiological stress can be a powerful tool for evaluating wildlife responses to diverse environmental changes or for monitoring changes in stress among individuals in free-ranging populations. Our study demonstrates the importance of careful study design and systematic sampling to ensure data are high quality and therefore useful for drawing meaningful ecological conclusions about wildlife stress.

## Materials and Methods

### Animals and housing

Fifteen wild-caught adult snowshoe hares were used in this experiment. Animals were captured in Lolo National Forest in Montana, USA (2 females, 3 males) and Mt. Baker-Snoqualmie National Forest in Washington, USA (4 females, 6 males). Animals were transferred from the field to an environmental facility at North Carolina State University (NCSU), College of Veterinary Medicine (CVM) and maintained in captivity for approximately one year prior to this experiment. Environmental rooms at this facility replicated site-specific temperature and photoperiod from the capture location of the animals. Animals were housed individually in wire mesh enclosures (121.9 cm width × 60.9 cm depth × 73.6 cm height) including a wire mesh floor with a waste collection tray below the wire floor, lined with absorbent paper. Enclosures included an acrylic hide box (30.5 cm width × 60.9 cm depth × 73.6 cm height) that functioned as a visual barrier between animals, as well as an edible grass mat and grass hut to hide in or sit atop. Water and premium natural adult rabbit food (Sherwood Pet Health, Logan, UT, USA) were available ad libitum. All capture, handling, transport, husbandry and experimental procedures were approved by North Carolina State University Institutional Animal Care and Use Committee (IACUC) and we confirm that all procedures were performed in accordance with approved IACUC protocol 14-069-0.

### Fecal sample collection

On evenings prior to pellet collection, we replaced the absorbent paper in the waste collection tray beneath each enclosure between 2145 and 2200 hours to provide a clean substrate for sampling. Then for six consecutive days, between 0600 and 0610 hours, we collected all fecal pellets beneath each animal’s enclosure, except pellets that were in contact with urine because urine may affect measured FGM concentrations^12^. We choose this eight-hour sampling frame to represent the time over which an animal may be held in a trap during field-based operations, which corresponds to the approximate lag time between stress-induced cortisol production and the expression of cortisol metabolites in snowshoe hares^28^. While this eight-hour sampling window could impact within-sample FGM homogeneity^32^, we previously quantified within-sample variation in snowshoe hare FGM and found that FGM is relatively homogeneously distributed within snowshoe hare fecal pellet groups accumulated over a similar sampling frame (2200-0800)^33^. As such, we placed each sample in an individually labeled bag and transferred it to a −20 °C freezer within 5 minutes of collection. Then, at the end of the collection period, we pooled each animal’s pellets and thoroughly mixed them, thereby creating a single large sample per individual. Each individual’s pooled sample was then subsequently divided into 25 equal portions based on sample wet weight. The pooled sample was necessary to obtain sufficient volume to be divided into 25 subsamples for the temperature and precipitation treatments and to ensure we obtained a sample representative of the average FGM concentration in pellets per individual as opposed to a random stress event, which could have unduly biased our FGM measurements.

### Experimental treatments

To assess the effect of age (max 6 days), all but one subsamples (n = 24) per individual were randomly assigned to one of 4 treatments for varying number of days. One subsample per individual was assigned to a pre-treatment control that was immediately returned to the −20 °C freezer. Environmental treatments included: (1) warm-dry [WD], (2) warm-wet [WW], (3) cool-dry [CD], and (4) cool-wet [CW]. Warm and cool temperatures represented average summer temperature in Washington and Montana and were based on monthly 1981–2010 normal temperatures in July and August at the snowshoe hare capture sites (mean warm = 23.5 °C; mean cool = 7.3 °C) (PRISM Climate Group). Similarly, summer precipitation was based on 1981–2010 normal precipitation data (PRISM Climate Group) during July and August (mean wet = 1 mm; dry = 0 mm). Accordingly, we applied 1 mm of precipitation daily at 0800 am via a hand-held spray bottle each day 24 hrs. prior to sample collection for the WW and CW treatments. For the experiment, all but the control samples were placed in a 17.78 cm × 17.78 cm soak resistant paper bowl with approximately 40 pine (*Pinus* sp.) needles broken into thirds to cover the bottom of the bowl. Samples were then subjected to an assigned environmental treatment (WD, WW, CD, CW) for up to 6 days. We collected samples at the end of each day treatment period and immediately placed the samples in a −20 °C freezer until FGM extractions.

### Extraction and quantification of fecal glucocorticoid metabolites

We thoroughly homogenized each sample using a glass mortar and pestle and then dried the samples at 80 °C for 48 hours^33^. After drying, we removed a 0.15 g subsample and placed it in 8.0 ml glass centrifuge tube with 5.0 ml of 80% methanol^16^. Next, we shook samples on a hand vortex for one minute and centrifuged each sample at 2,500 g for 15 minutes^16^. Following centrifugation, 0.5 ml of supernatant was transferred to micro-centrifuge tubes and dried for 2 hours at 80 °C^16^. Fecal extracts were then shipped to the Department of Biomedical Sciences, Unit of Physiology, Pathophysiology and Experimental Endocrinology at the University of Veterinary Medicine in Vienna, Austria for steroid analysis using an enzyme immunoassay (EIA)^33^. Concentrations of FGM were quantified in fecal extracts using an established 11-oxoetiocholanolone-EIA protocol^27^ validated for snowshoe hares^28^.

### Data analysis

Prior to analysis, we assessed the data for outliers by visually inspecting the raw values for each day and tested for normality and equal variances using diagnostic plots. We subsequently log-transformed FGM data to meet the assumption of normality and confirmed it using diagnostic plots. To determine the effects of fecal pellet age, temperature and precipitation on the FGM concentration, we fitted linear mixed effects models using the package lme4^34^. We included the fixed effects of age (days 1 through 6 of treatment exposure), precipitation (wet, dry) and temperature (warm, cool), and the random effect of individual hare to account for among-individual differences in FGM concentrations. In addition to linear terms, we included a quadratic term to allow for a potential curvilinear response of FGM concentrations to fecal pellets’ age. Additionally, we included a fixed effect of sex to examine its potential influence on the FGM concentrations.

Next, we tested the effect of pellets age on FGM concentrations to determine when FGM concentrations begin to vary within each environmental treatment relative to control FGM concentrations. We allocated the samples from the four different treatments into daily intervals (days 0 [i.e., control] through exposure day 6) and ran a simple mixed model for each treatment with a single fixed effect age coded as a factor and individual hares as a random effect. All statistical tests were conducted using R 3.3.2^35^ and significance of fixed effects were assessed with an alpha level of 0.05.

## Supplementary information

Dataset 1

## References

[1] Möstl E, Palme R (2002). Hormones as indicators of stress. Domestic animal endocrinology.

[2] Palme R, Rettenbacher S, Touma C, El-Bahr S, Möstl E (2005). Stress hormones in mammals and birds: comparative aspects regarding metabolism, excretion, and noninvasive measurement in fecal samples. Annals of the New York Academy of Sciences.

[3] Sheriff MJ, Dantzer B, Delehanty B, Palme R, Boonstra R (2011). Measuring stress in wildlife: techniques for quantifying glucocorticoids. Oecologia.

[4] Dantzer B, Fletcher QE, Boonstra R, Sheriff MJ (2014). Measures of physiological stress: a transparent or opaque window into the status, management and conservation of species?. Conservation Physiology.

[5] Millspaugh JJ, Washburn BE (2004). Use of fecal glucocorticoid metabolite measures in conservation biology research: considerations for application and interpretation. General and comparative endocrinology.

[6] Goymann W (2012). On the use of non‐invasive hormone research in uncontrolled, natural environments: the problem with sex, diet, metabolic rate and the individual. Methods in Ecology and Evolution.

[7] Palme R (2019). Non-invasive measurement of glucocorticoids: Advances and problems. Physiology & behavior.

[8] Harper JM, Austad SN (2000). Fecal glucocorticoids: a noninvasive method of measuring adrenal activity in wild and captive rodents. Physiological and Biochemical Zoology.

[9] Millspaugh, J. J. *et al*. Fecal glucocorticoid assays and the physiological stress response in elk. *Wildlife Society Bulletin*, 899–907 (2001).

[10] Touma C, Palme R (2005). Measuring fecal glucocorticoid metabolites in mammals and birds: the importance of validation. Annals of the New York Academy of Sciences.

[11] Madliger CL (2016). Success stories and emerging themes in conservation physiology. Conservation Physiology.

[12] Palme R (2005). Measuring fecal steroids: guidelines for practical application. Annals of the New York Academy of Sciences.

[13] Wasser SK (2000). A generalized fecal glucocorticoid assay for use in a diverse array of nondomestic mammalian and avian species. General and comparative endocrinology.

[14] Terio KA, Brown JL, Moreland R, Munson L (2002). Comparison of different drying and storage methods on quantifiable concentrations of fecal steroids in the cheetah. Zoo Biology.

[15] Washburn BE, Millspaugh JJ (2002). Effects of simulated environmental conditions on glucocorticoid metabolite measurements in white-tailed deer feces. General and comparative endocrinology.

[16] Palme R, Touma C, Arias N, Dominchin MF, Lepschy M (2013). Steroid extraction: get the best out of faecal samples. Wien Tierarztl Monatsschr.

[17] Wilkening JL, Ray C, Varner J (2016). When can we measure stress noninvasively? Postdeposition effects on a fecal stress metric confound a multiregional assessment. Ecology and evolution.

[18] Rehnus, M. Habitat selection of the Mountain hare (Lepus timidus) in the Central Alps. (na, 2009).

[19] Webster AB, Burroughs RE, Laver P, Ganswindt A (2018). Non-invasive assessment of adrenocortical activity as a measure of stress in leopards Panthera pardus. African Zoology.

[20] Creel S, Creel NM, Mills MG, Monfort SL (1997). Rank and reproduction in cooperatively breeding African wild dogs: behavioral and endocrine correlates. Behavioral Ecology.

[21] Creel S (2002). Snowmobile activity and glucocorticoid stress responses in wolves and elk. Conservation Biology.

[22] Creel S, Winnie JA, Christianson D (2009). Glucocorticoid stress hormones and the effect of predation risk on elk reproduction. Proceedings of the National Academy of Sciences.

[23] Zimova M, Mills LS, Nowak JJ (2016). High fitness costs of climate change‐induced camouflage mismatch. Ecology letters.

[24] Mills LS (2018). Winter color polymorphisms identify global hot spots for evolutionary rescue from climate change. Science.

[25] Mills LS (2013). Camouflage mismatch in seasonal coat color due to decreased snow duration. Proceedings of the National Academy of Sciences.

[26] Jones MR (2018). Adaptive introgression underlies polymorphic seasonal camouflage in snowshoe hares. Science.

[27] Palme, R. & Möstl, E. Measurement of cortisol metabolites in faeces of sheep as a parameter of cortisol concentration in blood. *Zeitschrift fuer Saeugetierkunde (Germany)* (1997).

[28] Sheriff MJ, Bosson CO, Krebs CJ, Boonstra R (2009). A non-invasive technique for analyzing fecal cortisol metabolites in snowshoe hares (Lepus americanus). Journal of Comparative Physiology B.

[29] Millspaugh, J. J., Washburn, B. E., Milanick, M. A., Slotow, R. & van Dyk, G. Effects of heat and chemical treatments on fecal glucocorticoid measurements: implications for sample transport. *Wildlife Society Bulletin*, 399–406 (2003).

[30] Stetz J, Hunt K, Kendall KC, Wasser SK (2013). Effects of exposure, diet, and thermoregulation on fecal glucocorticoid measures in wild bears. PloS one.

[31] Cheng E, Hodges KE, Sollmann R, Mills LS (2017). Genetic sampling for estimating density of common species. Ecology and evolution.

[32] Ganswindt A, Tordiffe ASW, Stam E, Howitt M, Jori F (2012). Determining adrenocortical activity as a measure of stress in African buffalo (Syncerus caffer) based on faecal analysis. African Zoology.

[33] Lafferty DJ, Kumar AV, Whitcher S, Hackländer K, Mills LS (2017). Within-sample variation in snowshoe hare faecal glucocorticoid metabolite measurements. Conservation physiology.

[34] Bates, D., Maechler, M., Bolker, B. & Walker, S. lme4: Linear mixed-effects models using Eigen and S4. R package version 1.1-7. This is computer program (R package). The URL of the package is: http://CRAN.R-project.org/package=lme4 (2014).

[35] Team, R. C. R: A language and environment for statistical computing. R Foundation for Statistical Computing, Vienna, Austria. 2015, http.www.R-project.org (2016).

