# Supplementary material for: Noninvasive measures of physiological stress are confounded by exposure
Source: Sci Rep. 2019 Dec 16;9:19170. doi: 10.1038/s41598-019-55715-5 (PMC6915565; doi:10.1038/s41598-019-55715-5)
Supplement: Supplementary file 1 — Dataset 1 [file 41598_2019_55715_MOESM1_ESM.pdf]

Noninvasive measures of physiological stress are confounded by exposure

Diana J. R. Lafferty[abc‡*], Marketa Zimova[cd‡], Lindsay Clontz[b], Klaus Hackländer[e], L. Scott Mills[cf]

[a] Wildlife Ecology and Conservation Science Lab, Department of Biology, Northern Michigan University, Marquette, MI 49855, USA

[b] Fisheries, Wildlife, and Conservation Biology Program, Department of Forestry and Environmental Resources, North Carolina State University, Raleigh, NC 27695, USA

[c] Wildlife Biology Program, University of Montana, Missoula, MT 59812, USA

[d] School for Environmental and Sustainability, Institute for Global Change Biology, University of Michigan, Ann Arbor, MI 48104

[e] Institute of Wildlife Biology and Game Management, University of Natural Resources and Life Sciences, Vienna, Gregor-Mendel-Str. 33, 1180 Vienna, Austria

[f] Office of Research and Creative Scholarship, University of Montana, Missoula, MT 59812, USA

[‡] These authors contributed equally to this work.

[*]Corresponding author: fax: +1-906-227-1063, tel: +1-906-227-2227, dlaffert@nmu.edu

sample	indiv	sex	total.weight	indiv.weight	wet.weight	dry.weight	pg	ng	temp	precip	time	trt	trt_time	state
292	Arthur	M	275.6	11	9.343	9.154	38.5	84.7	S	D	1	SD	SD_1	WA
288	Avery	M	213.6	8.5	9.664	9.478	21.4	47	S	D	1	SD	SD_1	WA
297	Bunn	M	346.6	13.9	9.877	9.706	10.8	23.8	S	D	1	SD	SD_1	MT
293	Caer	F	333.6	13.3	9.336	9.147	33.8	74.3	S	D	1	SD	SD_1	WA
287	Cortana	F	314.6	12.6	9.006	9.689	20.6	45.2	S	D	1	SD	SD_1	WA
300	David	M	405.6	16.2	9.889	9.669	27.6	60.6	S	D	1	SD	SD_1	MT
291	Herbert	M	360.6	14.4	9.31	8.893	35.7	78.5	S	D	1	SD	SD_1	WA
298	Hypnotod	F	688.6	27.5	9.376	9.654	39.7	87.3	S	D	1	SD	SD_1	MT
286	Jacob	M	349.6	14	9.333	9.158	34	74.9	S	D	1	SD	SD_1	WA
294	Lance	M	185.6	7.4	9.581	9.424	10.6	23.3	S	D	1	SD	SD_1	WA
289	Master Chief	M	286.6	11.5	9.129	8.966	19.4	42.7	S	D	1	SD	SD_1	WA
296	Nibbler	F	394.6	15.8	10.303	10.074	19.1	42	S	D	1	SD	SD_1	MT
299	Pandoodlek	F	463.6	18.5	9.658	9.283	38.5	84.6	S	D	1	SD	SD_1	MT
295	Robin	M	245.6	9.8	10.009	9.829	37.6	82.7	S	D	1	SD	SD_1	WA
290	Zoot	F	254.6	10.2	9.564	9.407	20.1	44.3	S	D	1	SD	SD_1	WA
202	Arthur	M	275.6	11	9.127	9.981	16	35.1	S	W	1	SW	SW_1	WA
198	Avery	M	213.6	8.5	9.075	8.941	35	76.9	S	W	1	SW	SW_1	WA
207	Bunn	M	346.6	13.9	9.889	9.693	4	8.8	S	W	1	SW	SW_1	MT
203	Caer	F	333.6	13.3	9.458	9.318	13.7	30.2	S	W	1	SW	SW_1	WA
197	Cortana	F	314.6	12.6	9.218	9.063	2.7	5.9	S	W	1	SW	SW_1	WA
210	David	M	405.6	16.2	9.4	9.222	17.7	39	S	W	1	SW	SW_1	MT
201	Herbert	M	360.6	14.4	9.215	9.029	29.1	64	S	W	1	SW	SW_1	WA
208	Hypnotod	F	688.6	27.5	9.495	9.043	27.6	60.7	S	W	1	SW	SW_1	MT
196	Jacob	M	349.6	14	9.469	9.294	4.7	10.4	S	W	1	SW	SW_1	WA
204	Lance	M	185.6	7.4	9.057	8.93	6.5	14.3	S	W	1	SW	SW_1	WA
199	Master Chief	M	286.6	11.5	9.579	9.405	13.1	28.7	S	W	1	SW	SW_1	WA
206	Nibbler	F	394.6	15.8	9.908	9.705	4.7	10.3	S	W	1	SW	SW_1	MT
209	Pandoodlek	F	463.6	18.5	9.223	8.907	30.3	66.7	S	W	1	SW	SW_1	MT
205	Robin	M	245.6	9.8	9.815	9.625	8.2	18	S	W	1	SW	SW_1	WA
200	Zoot	F	254.6	10.2	9.411	9.279	13.2	29	S	W	1	SW	SW_1	WA
112	Arthur	M	275.6	11	10.429	9.205	13.4	29.4	W	D	1	WD	WD_1	WA
108	Avery	M	213.6	8.5	9.208	8.553	33.6	73.9	W	D	1	WD	WD_1	WA
117	Bunn	M	346.6	13.9	10.441	9.725	12.8	28.2	W	D	1	WD	WD_1	MT
113	Caer	F	333.6	13.3	10.735	9.519	26.4	58.1	W	D	1	WD	WD_1	WA
107	Cortana	F	314.6	12.6	10.07	9.126	22.3	49.1	W	D	1	WD	WD_1	WA
120	David	M	405.6	16.2	10.052	9.176	10.7	23.6	W	D	1	WD	WD_1	MT
111	Herbert	M	360.6	14.4	10.074	8.816	17.4	38.4	W	D	1	WD	WD_1	WA
118	Hypnotod	F	688.6	27.5	11.565	9.779	23.6	51.8	W	D	1	WD	WD_1	MT
106	Jacob	M	349.6	14	10.46	9.528	27.2	59.7	W	D	1	WD	WD_1	WA
114	Lance	M	185.6	7.4	9.994	9.552	7.7	16.8	W	D	1	WD	WD_1	WA
109	Master Chief	M	286.6	11.5	9.108	8.641	15.5	34.1	W	D	1	WD	WD_1	WA
116	Nibbler	F	394.6	15.8	10.457	9.587	7.3	16	W	D	1	WD	WD_1	MT
119	Pandoodlek	F	463.6	18.5	10.781	9.387	26.4	58.1	W	D	1	WD	WD_1	MT

115	Robin	M	245.6	9.8	10.146	9.583	5.8	12.8	W	D	1	WD	WD_1	WA
110	Zoot	F	254.6	10.2	10.125	9.573	19	41.8	W	D	1	WD	WD_1	WA
22	Arthur	M	275.6	11	9.461	8.816	13.5	29.8	W	W	1	WW	WW_1	WA
18	Avery	M	213.6	8.5	9.621	9.131	26.6	58.5	W	W	1	WW	WW_1	WA
27	Bunn	M	346.6	13.9	10.235	9.344	1	2.2	W	W	1	WW	WW_1	MT
23	Caer	F	333.6	13.3	9.604	8.895	14.6	32.1	W	W	1	WW	WW_1	WA
17	Cortana	F	314.6	12.6	9.745	9.244	10.6	23.3	W	W	1	WW	WW_1	WA
30	David	M	405.6	16.2	9.827	8.98	10.5	23	W	W	1	WW	WW_1	MT
21	Herbert	M	360.6	14.4	10.028	8.916	17.6	38.7	W	W	1	WW	WW_1	WA
28	Hypnotod	F	688.6	27.5	10.661	9.13	38.9	85.7	W	W	1	WW	WW_1	MT
16	Jacob	M	349.6	14	9.737	9.07	7.3	16	W	W	1	WW	WW_1	WA
24	Lance	M	185.6	7.4	9.693	9.329	1	2.2	W	W	1	WW	WW_1	WA
19	Master Chief	M	286.6	11.5	9.6	9.164	10.3	22.7	W	W	1	WW	WW_1	WA
26	Nibbler	F	394.6	15.8	10.172	9.214	18.6	40.9	W	W	1	WW	WW_1	MT
29	Pandoodlek	F	463.6	18.5	10.139	9.163	22.7	49.9	W	W	1	WW	WW_1	MT
25	Robin	M	245.6	9.8	9.602	9.191	1	2.2	W	W	1	WW	WW_1	WA
20	Zoot	F	254.6	10.2	9.698	9.334	16.1	35.4	W	W	1	WW	WW_1	WA
307	Arthur	M	275.6	11	9.664	9.189	18.7	41.1	S	D	2	SD	SD_2	WA
303	Avery	M	213.6	8.5	9.679	9.535	20.1	44.2	S	D	2	SD	SD_2	WA
312	Bunn	M	346.6	13.9	9.843	9.695	11.2	24.6	S	D	2	SD	SD_2	MT
308	Caer	F	333.6	13.3	9.225	9.1	28.9	63.5	S	D	2	SD	SD_2	WA
302	Cortana	F	314.6	12.6	9.396	9.258	14.1	31	S	D	2	SD	SD_2	WA
315	David	M	405.6	16.2	9.793	9.655	16.3	35.9	S	D	2	SD	SD_2	MT
306	Herbert	M	360.6	14.4	9.268	9.139	27.6	60.7	S	D	2	SD	SD_2	WA
313	Hypnotod	F	688.6	27.5	9.443	9.314	55.3	121.6	S	D	2	SD	SD_2	MT
301	Jacob	M	349.6	14	9.725	9.572	32.3	71	S	D	2	SD	SD_2	WA
309	Lance	M	185.6	7.4	9.767	9.609	14.8	32.5	S	D	2	SD	SD_2	WA
304	Master Chief	M	286.6	11.5	9.434	9.311	16.3	35.8	S	D	2	SD	SD_2	WA
311	Nibbler	F	394.6	15.8	9.675	9.536	11	24.2	S	D	2	SD	SD_2	MT
314	Pandoodlek	F	463.6	18.5	9.605	9.474	42.2	92.8	S	D	2	SD	SD_2	MT
310	Robin	M	245.6	9.8	10.113	9.919	25.3	55.6	S	D	2	SD	SD_2	WA
305	Zoot	F	254.6	10.2	9.675	9.52	27.1	59.6	S	D	2	SD	SD_2	WA
217	Arthur	M	275.6	11	9.682	9.533	24	52.8	S	W	2	SW	SW_2	WA
213	Avery	M	213.6	8.5	9.265	9.155	15.4	33.9	S	W	2	SW	SW_2	WA
222	Bunn	M	346.6	13.9	9.346	9.214	7.8	17.2	S	W	2	SW	SW_2	MT
218	Caer	F	333.6	13.3	9.515	9.39	41	90.2	S	W	2	SW	SW_2	WA
212	Cortana	F	314.6	12.6	9.544	9.4	16.2	35.6	S	W	2	SW	SW_2	WA
225	David	M	405.6	16.2	9.092	8.973	23	50.6	S	W	2	SW	SW_2	MT
216	Herbert	M	360.6	14.4	9.233	9.1	26.7	58.8	S	W	2	SW	SW_2	WA
223	Hypnotod	F	688.6	27.5	9.221	9.091	15.1	33.3	S	W	2	SW	SW_2	MT
211	Jacob	M	349.6	14	9.708	9.567	14.7	32.3	S	W	2	SW	SW_2	WA
219	Lance	M	185.6	7.4	9.111	8.971	4.5	9.9	S	W	2	SW	SW_2	WA
214	Master Chief	M	286.6	11.5	9.263	9.126	17.8	39.2	S	W	2	SW	SW_2	WA
221	Nibbler	F	394.6	15.8	9.642	9.5	7.6	16.7	S	W	2	SW	SW_2	MT

224	Pandoodlek	F	463.6	18.5	9.479	9.354	42.7	93.8	S	W	2	SW	SW_2	MT
220	Robin	M	245.6	9.8	9.833	9.767	10.8	23.7	S	W	2	SW	SW_2	WA
215	Zoot	F	254.6	10.2	9.231	9.109	28.4	62.4	S	W	2	SW	SW_2	WA
127	Arthur	M	275.6	11	10.03	9.022	14.9	32.7	W	D	2	WD	WD_2	WA
123	Avery	M	213.6	8.5	9.308	8.978	18.3	40.2	W	D	2	WD	WD_2	WA
132	Bunn	M	346.6	13.9	9.853	9.404	5.6	12.4	W	D	2	WD	WD_2	MT
128	Caer	F	333.6	13.3	9.721	9.184	14.4	31.7	W	D	2	WD	WD_2	WA
122	Cortana	F	314.6	12.6	9.86	9.353	13.4	29.5	W	D	2	WD	WD_2	WA
135	David	M	405.6	16.2	9.781	9.397	13.9	30.5	W	D	2	WD	WD_2	MT
126	Herbert	M	360.6	14.4	9.604	8.899	24.9	54.8	W	D	2	WD	WD_2	WA
133	Hypnotod	F	688.6	27.5	10.668	9.535	58.4	128.6	W	D	2	WD	WD_2	MT
121	Jacob	M	349.6	14	9.313	8.905	20.8	45.8	W	D	2	WD	WD_2	WA
129	Lance	M	185.6	7.4	9.738	9.437	14.3	31.5	W	D	2	WD	WD_2	WA
124	Master Chief	M	286.6	11.5	9.882	9.403	3.2	7.1	W	D	2	WD	WD_2	WA
131	Nibbler	F	394.6	15.8	10.113	9.733	13.4	29.5	W	D	2	WD	WD_2	MT
134	Pandoodlek	F	463.6	18.5	9.751	9.262	27.3	60.1	W	D	2	WD	WD_2	MT
130	Robin	M	245.6	9.8	9.548	9.144	23.7	52.2	W	D	2	WD	WD_2	WA
125	Zoot	F	254.6	10.2	9.586	9.22	24.2	53.2	W	D	2	WD	WD_2	WA
37	Arthur	M	275.6	11	9.833	9.311	25.8	56.7	W	W	2	WW	WW_2	WA
33	Avery	M	213.6	8.5	9.2	8.909	22.6	49.7	W	W	2	WW	WW_2	WA
42	Bunn	M	346.6	13.9	10.107	9.738	8.2	18	W	W	2	WW	WW_2	MT
38	Caer	F	333.6	13.3	9.256	8.878	26.2	57.6	W	W	2	WW	WW_2	WA
32	Cortana	F	314.6	12.6	9.344	9.017	9.2	20.2	W	W	2	WW	WW_2	WA
45	David	M	405.6	16.2	9.282	8.793	22.9	50.3	W	W	2	WW	WW_2	MT
36	Herbert	M	360.6	14.4	9.538	9.05	19.3	42.5	W	W	2	WW	WW_2	WA
43	Hypnotod	F	688.6	27.5	9.47	8.959	25.7	56.6	W	W	2	WW	WW_2	MT
31	Jacob	M	349.6	14	9.351	8.987	22.2	48.9	W	W	2	WW	WW_2	WA
39	Lance	M	185.6	7.4	9.451	9.186	9.1	20	W	W	2	WW	WW_2	WA
34	Master Chief	M	286.6	11.5	9.624	9.172	28.3	62.3	W	W	2	WW	WW_2	WA
41	Nibbler	F	394.6	15.8	9.874	9.444	24.2	53.3	W	W	2	WW	WW_2	MT
44	Pandoodlek	F	463.6	18.5	9.833	9.334	50.8	111.8	W	W	2	WW	WW_2	MT
40	Robin	M	245.6	9.8	9.953	9.592	26.8	58.9	W	W	2	WW	WW_2	WA
35	Zoot	F	254.6	10.2	9.674	9.392	29.1	64	W	W	2	WW	WW_2	WA
322	Arthur	M	275.6	11	9.754	9.606	35.8	78.7	S	D	3	SD	SD_3	WA
318	Avery	M	213.6	8.5	9.274	9.142	21.7	47.8	S	D	3	SD	SD_3	WA
327	Bunn	M	346.6	13.9	9.746	9.607	13.4	29.5	S	D	3	SD	SD_3	MT
323	Caer	F	333.6	13.3	9.441	9.316	39.6	87.1	S	D	3	SD	SD_3	WA
317	Cortana	F	314.6	12.6	9.216	9.093	16.4	36	S	D	3	SD	SD_3	WA
330	David	M	405.6	16.2	9.35	9.229	21.3	46.9	S	D	3	SD	SD_3	MT
321	Herbert	M	360.6	14.4	9.558	9.435	56.4	124	S	D	3	SD	SD_3	WA
328	Hypnotod	F	688.6	27.5	9.376	9.248	27.2	59.8	S	D	3	SD	SD_3	MT
316	Jacob	M	349.6	14	9.555	9.422	15.1	33.2	S	D	3	SD	SD_3	WA
324	Lance	M	185.6	7.4	9.489	9.378	11	24.1	S	D	3	SD	SD_3	WA
319	Master Chief	M	286.6	11.5	9.451	9.321	29.8	65.5	S	D	3	SD	SD_3	WA

326	Nibbler	F	394.6	15.8	9.953	8.16	25.9	56.9	S	D	3	SD	SD_3	MT
329	Pandoodlek	F	463.6	18.5	9.43	9.294	21.7	47.8	S	D	3	SD	SD_3	MT
325	Robin	M	245.6	9.8	9.903	9.755	21.2	46.6	S	D	3	SD	SD_3	WA
320	Zoot	F	254.6	10.2	9.291	9.156	37.4	82.2	S	D	3	SD	SD_3	WA
232	Arthur	M	275.6	11	9.379	9.266	11.2	24.7	S	W	3	SW	SW_3	WA
228	Avery	M	213.6	8.5	9.593	9.436	26	57.3	S	W	3	SW	SW_3	WA
237	Bunn	M	346.6	13.9	9.893	9.765	9.5	21	S	W	3	SW	SW_3	MT
233	Caer	F	333.6	13.3	9.531	9.356	15.1	33.2	S	W	3	SW	SW_3	WA
227	Cortana	F	314.6	12.6	9.466	9.339	8.6	18.9	S	W	3	SW	SW_3	WA
240	David	M	405.6	16.2	9.398	9.261	14.1	31	S	W	3	SW	SW_3	MT
231	Herbert	M	360.6	14.4	9.396	9.235	29	63.8	S	W	3	SW	SW_3	WA
238	Hypnotod	F	688.6	27.5	9.952	9.815	29	63.8	S	W	3	SW	SW_3	MT
226	Jacob	M	349.6	14	9.696	9.535	14.3	31.4	S	W	3	SW	SW_3	WA
234	Lance	M	185.6	7.4	9.776	9.605	6.3	13.9	S	W	3	SW	SW_3	WA
229	Master Chief	M	286.6	11.5	9.203	9.058	14.3	31.4	S	W	3	SW	SW_3	WA
236	Nibbler	F	394.6	15.8	9.607	9.462	6.7	14.7	S	W	3	SW	SW_3	MT
239	Pandoodlek	F	463.6	18.5	9.887	9.716	41.1	90.4	S	W	3	SW	SW_3	MT
235	Robin	M	245.6	9.8	9.417	9.269	11.9	26.1	S	W	3	SW	SW_3	WA
230	Zoot	F	254.6	10.2	9.642	9.508	31.3	68.9	S	W	3	SW	SW_3	WA
142	Arthur	M	275.6	11	9.977	9.644	31.3	68.9	W	D	3	WD	WD_3	WA
138	Avery	M	213.6	8.5	8.679	8.455	39	85.8	W	D	3	WD	WD_3	WA
147	Bunn	M	346.6	13.9	9.797	9.52	1	2.2	W	D	3	WD	WD_3	MT
143	Caer	F	333.6	13.3	9.88	9.482	46.7	102.7	W	D	3	WD	WD_3	WA
137	Cortana	F	314.6	12.6	9.83	9.505	25.8	56.7	W	D	3	WD	WD_3	WA
150	David	M	405.6	16.2	9.44	9.147	26.7	58.7	W	D	3	WD	WD_3	MT
141	Herbert	M	360.6	14.4	9.748	9.408	40.9	90	W	D	3	WD	WD_3	WA
148	Hypnotod	F	688.6	27.5	10.131	9.576	30.8	67.8	W	D	3	WD	WD_3	MT
136	Jacob	M	349.6	14	9.64	9.295	22.4	49.2	W	D	3	WD	WD_3	WA
144	Lance	M	185.6	7.4	9.377	9.146	13.3	29.3	W	D	3	WD	WD_3	WA
139	Master Chief	M	286.6	11.5	9.668	9.315	21.6	47.6	W	D	3	WD	WD_3	WA
146	Nibbler	F	394.6	15.8	10.04	9.705	14.1	31.1	W	D	3	WD	WD_3	MT
149	Pandoodlek	F	463.6	18.5	9.937	9.603	36.8	81	W	D	3	WD	WD_3	MT
145	Robin	M	245.6	9.8	10.042	9.352	15.7	34.4	W	D	3	WD	WD_3	WA
140	Zoot	F	254.6	10.2	9.831	9.529	41.4	91.1	W	D	3	WD	WD_3	WA
52	Arthur	M	275.6	11	9.083	8.804	20.7	45.6	W	W	3	WW	WW_3	WA
48	Avery	M	213.6	8.5	9.388	9.084	27.6	60.8	W	W	3	WW	WW_3	WA
57	Bunn	M	346.6	13.9	9.723	9.351	18.9	41.6	W	W	3	WW	WW_3	MT
53	Caer	F	333.6	13.3	9.802	9.453	30.1	66.2	W	W	3	WW	WW_3	WA
47	Cortana	F	314.6	12.6	9.581	9.177	16.4	36	W	W	3	WW	WW_3	WA
60	David	M	405.6	16.2	10.003	9.526	16.3	35.9	W	W	3	WW	WW_3	MT
51	Herbert	M	360.6	14.4	9.698	9.197	25.2	55.5	W	W	3	WW	WW_3	WA
58	Hypnotod	F	688.6	27.5	9.799	9.107	37.3	82	W	W	3	WW	WW_3	MT
46	Jacob	M	349.6	14	9.633	9.276	14.1	31	W	W	3	WW	WW_3	WA
54	Lance	M	185.6	7.4	9.499	9.219	10.8	23.7	W	W	3	WW	WW_3	WA

49	Master Chief	M	286.6	11.5	9.25	8.983	26.4	58	W	W	3	WW	WW_3	WA
56	Nibbler	F	394.6	15.8	9.2	8.862	14.3	31.5	W	W	3	WW	WW_3	MT
59	Pandoodlek	F	463.6	18.5	9.729	9.264	33	72.7	W	W	3	WW	WW_3	MT
55	Robin	M	245.6	9.8	9.764	9.415	21.9	48.2	W	W	3	WW	WW_3	WA
50	Zoot	F	254.6	10.2	9.397	9.094	29	63.9	W	W	3	WW	WW_3	WA
337	Arthur	M	275.6	11	9.681	9.552	21.1	46.5	S	D	4	SD	SD_4	WA
333	Avery	M	213.6	8.5	9.406	9.272	25.4	55.9	S	D	4	SD	SD_4	WA
342	Bunn	M	346.6	13.9	9.572	9.423	17.1	37.5	S	D	4	SD	SD_4	MT
338	Caer	F	333.6	13.3	9.371	9.627	27	59.4	S	D	4	SD	SD_4	WA
332	Cortana	F	314.6	12.6	9.431	9.307	16.8	37	S	D	4	SD	SD_4	WA
345	David	M	405.6	16.2	9.387	9.266	19.7	43.3	S	D	4	SD	SD_4	MT
336	Herbert	M	360.6	14.4	9.204	9.075	59.2	130.3	S	D	4	SD	SD_4	WA
343	Hypnotod	F	688.6	27.5	9.489	9.369	21.1	46.5	S	D	4	SD	SD_4	MT
331	Jacob	M	349.6	14	9.564	9.503	15.5	34	S	D	4	SD	SD_4	WA
339	Lance	M	185.6	7.4	9.459	9.353	1	2.2	S	D	4	SD	SD_4	WA
334	Master Chief	M	286.6	11.5	9.492	9.38	25.5	56	S	D	4	SD	SD_4	WA
341	Nibbler	F	394.6	15.8	9.682	9.541	17	37.3	S	D	4	SD	SD_4	MT
344	Pandoodlek	F	463.6	18.5	9.689	9.5726	43.1	94.8	S	D	4	SD	SD_4	MT
340	Robin	M	245.6	9.8	9.966	9.806	28.5	62.7	S	D	4	SD	SD_4	WA
335	Zoot	F	254.6	10.2	9.701	9.563	19.6	43.1	S	D	4	SD	SD_4	WA
247	Arthur	M	275.6	11	9.648	9.423	20.2	44.4	S	W	4	SW	SW_4	WA
243	Avery	M	213.6	8.5	9.99	9.442	26.9	59.2	S	W	4	SW	SW_4	WA
252	Bunn	M	346.6	13.9	9.497	9.332	11.3	24.8	S	W	4	SW	SW_4	MT
248	Caer	F	333.6	13.3	9.55	9.383	38.1	83.8	S	W	4	SW	SW_4	WA
242	Cortana	F	314.6	12.6	9.437	9.286	20.6	45.4	S	W	4	SW	SW_4	WA
255	David	M	405.6	16.2	9.324	9.173	14.7	32.2	S	W	4	SW	SW_4	MT
246	Herbert	M	360.6	14.4	9.855	9.675	28	61.7	S	W	4	SW	SW_4	WA
253	Hypnotod	F	688.6	27.5	9.488	9.321	33.7	74.2	S	W	4	SW	SW_4	MT
241	Jacob	M	349.6	14	9.526	9.364	22.7	49.9	S	W	4	SW	SW_4	WA
249	Lance	M	185.6	7.4	9.449	9.285	12.3	27.1	S	W	4	SW	SW_4	WA
244	Master Chief	M	286.6	11.5	9.752	9.573	16.6	36.6	S	W	4	SW	SW_4	WA
251	Nibbler	F	394.6	15.8	9.387	9.235	20.3	44.7	S	W	4	SW	SW_4	MT
254	Pandoodlek	F	463.6	18.5	9.681	9.506	25.6	56.4	S	W	4	SW	SW_4	MT
250	Robin	M	245.6	9.8	10.275	10.06	25.2	55.4	S	W	4	SW	SW_4	WA
245	Zoot	F	254.6	10.2	9.653	9.496	37.2	81.8	S	W	4	SW	SW_4	WA
157	Arthur	M	275.6	11	9.413	9.125	25.6	56.2	W	D	4	WD	WD_4	WA
153	Avery	M	213.6	8.5	9.942	9.658	31.3	68.9	W	D	4	WD	WD_4	WA
162	Bunn	M	346.6	13.9	9.416	0.143	8.5	18.7	W	D	4	WD	WD_4	MT
158	Caer	F	333.6	13.3	9.801	9.513	43.3	95.2	W	D	4	WD	WD_4	WA
152	Cortana	F	314.6	12.6	9.594	9.319	1	2.2	W	D	4	WD	WD_4	WA
165	David	M	405.6	16.2	9.433	9.143	8.5	18.8	W	D	4	WD	WD_4	MT
156	Herbert	M	360.6	14.4	9.397	9.104	40.2	88.5	W	D	4	WD	WD_4	WA
163	Hypnotod	F	688.6	27.5	9.761	9.385	21.8	48	W	D	4	WD	WD_4	MT
151	Jacob	M	349.6	14	9.436	9.168	15	32.9	W	D	4	WD	WD_4	WA

159	Lance	M	185.6	7.4	9.979	9.691	4.3	9.4	W	D	4	WD	WD_4	WA
154	Master Chief	M	286.6	11.5	9.826	9.543	25.1	55.2	W	D	4	WD	WD_4	WA
161	Nibbler	F	394.6	15.8	9.878	9.554	20.3	44.7	W	D	4	WD	WD_4	MT
164	Pandoodlek	F	463.6	18.5	9.365	9.062	24.2	53.3	W	D	4	WD	WD_4	MT
160	Robin	M	245.6	9.8	10.293	9.95	14	30.8	W	D	4	WD	WD_4	WA
155	Zoot	F	254.6	10.2	10.377	10.045	31.7	69.7	W	D	4	WD	WD_4	WA
67	Arthur	M	275.6	11	9.447	9.151	27.2	59.9	W	W	4	WW	WW_4	WA
63	Avery	M	213.6	8.5	9.009	8.786	36.2	79.5	W	W	4	WW	WW_4	WA
72	Bunn	M	346.6	13.9	9.686	9.352	14	30.7	W	W	4	WW	WW_4	MT
68	Caer	F	333.6	13.3	9.541	9.256	26.8	59	W	W	4	WW	WW_4	WA
62	Cortana	F	314.6	12.6	9.541	9.219	20.3	44.6	W	W	4	WW	WW_4	WA
75	David	M	405.6	16.2	9.532	8.979	14.8	32.5	W	W	4	WW	WW_4	MT
66	Herbert	M	360.6	14.4	9.484	9.053	29.1	64	W	W	4	WW	WW_4	WA
73	Hypnotod	F	688.6	27.5	9.916	9.327	22.6	49.6	W	W	4	WW	WW_4	MT
61	Jacob	M	349.6	14	9.195	8.892	22.8	50.1	W	W	4	WW	WW_4	WA
69	Lance	M	185.6	7.4	9.131	8.905	16.6	36.5	W	W	4	WW	WW_4	WA
64	Master Chief	M	286.6	11.5	9.214	8.93	19.2	42.3	W	W	4	WW	WW_4	WA
71	Nibbler	F	394.6	15.8	9.646	9.312	10.3	22.7	W	W	4	WW	WW_4	MT
74	Pandoodlek	F	463.6	18.5	9.268	8.894	44.7	98.4	W	W	4	WW	WW_4	MT
70	Robin	M	245.6	9.8	9.903	9.535	25.4	55.8	W	W	4	WW	WW_4	WA
65	Zoot	F	254.6	10.2	9.623	9.357	30.5	67.2	W	W	4	WW	WW_4	WA
352	Arthur	M	275.6	11	9.662	9.512	27.8	61.1	S	D	5	SD	SD_5	WA
348	Avery	M	213.6	8.5	9.433	9.288	35.4	77.9	S	D	5	SD	SD_5	WA
357	Bunn	M	346.6	13.9	9.627	9.489	1	2.2	S	D	5	SD	SD_5	MT
353	Caer	F	333.6	13.3	10.054	9.854	6.5	14.4	S	D	5	SD	SD_5	WA
347	Cortana	F	314.6	12.6	9.392	9.25	1	2.2	S	D	5	SD	SD_5	WA
360	David	M	405.6	16.2	9.517	9.393	34	74.9	S	D	5	SD	SD_5	MT
351	Herbert	M	360.6	14.4	8.958	8.845	20.5	45.2	S	D	5	SD	SD_5	WA
358	Hypnotod	F	688.6	27.5	9.581	9.449	27.4	60.3	S	D	5	SD	SD_5	MT
346	Jacob	M	349.6	14	9.319	9.192	21.9	48.1	S	D	5	SD	SD_5	WA
354	Lance	M	185.6	7.4	9.623	9.468	1	2.2	S	D	5	SD	SD_5	WA
349	Master Chief	M	286.6	11.5	9.488	9.365	10.4	23	S	D	5	SD	SD_5	WA
356	Nibbler	F	394.6	15.8	9.808	9.648	19.3	42.5	S	D	5	SD	SD_5	MT
359	Pandoodlek	F	463.6	18.5	9.939	9.79	38.6	84.9	S	D	5	SD	SD_5	MT
355	Robin	M	245.6	9.8	10.571	10.407	8.7	19.1	S	D	5	SD	SD_5	WA
350	Zoot	F	254.6	10.2	9.802	9.653	9.4	20.6	S	D	5	SD	SD_5	WA
262	Arthur	M	275.6	11	9.63	9.47	14.1	30.9	S	W	5	SW	SW_5	WA
258	Avery	M	213.6	8.5	9.594	9.44	23.9	52.6	S	W	5	SW	SW_5	WA
267	Bunn	M	346.6	13.9	9.444	9.296	2.6	5.7	S	W	5	SW	SW_5	MT
263	Caer	F	333.6	13.3	9.3	9.154	29.3	64.4	S	W	5	SW	SW_5	WA
257	Cortana	F	314.6	12.6	9.258	9.126	11.3	24.9	S	W	5	SW	SW_5	WA
270	David	M	405.6	16.2	9.45	9.328	36.5	80.2	S	W	5	SW	SW_5	MT
261	Herbert	M	360.6	14.4	8.906	8.776	25.4	55.8	S	W	5	SW	SW_5	WA
268	Hypnotod	F	688.6	27.5	9.496	9.323	18.2	40	S	W	5	SW	SW_5	MT

256	Jacob	M	349.6	14	9.548	9.404	11.5	25.4	S	W	5	SW	SW_5	WA
264	Lance	M	185.6	7.4	9.278	9.138	7	15.3	S	W	5	SW	SW_5	WA
259	Master Chief	M	286.6	11.5	9.379	9.247	10.1	22.2	S	W	5	SW	SW_5	WA
266	Nibbler	F	394.6	15.8	9.952	9.793	16.7	36.6	S	W	5	SW	SW_5	MT
269	Pandoodlek	F	463.6	18.5	9.341	9.203	23.7	52.1	S	W	5	SW	SW_5	MT
265	Robin	M	245.6	9.8	10.272	10.083	7.8	17.1	S	W	5	SW	SW_5	WA
260	Zoot	F	254.6	10.2	9.478	9.323	15.6	34.2	S	W	5	SW	SW_5	WA
172	Arthur	M	275.6	11	9.524	9.264	20.8	45.7	W	D	5	WD	WD_5	WA
168	Avery	M	213.6	8.5	9.815	9.539	26.8	59	W	D	5	WD	WD_5	WA
177	Bunn	M	346.6	13.9	9.717	9.455	8.8	19.4	W	D	5	WD	WD_5	MT
173	Caer	F	333.6	13.3	9.538	9.251	36.4	80.2	W	D	5	WD	WD_5	WA
167	Cortana	F	314.6	12.6	9.235	8.99	8.2	18	W	D	5	WD	WD_5	WA
180	David	M	405.6	16.2	9.838	9.562	29.7	65.2	W	D	5	WD	WD_5	MT
171	Herbert	M	360.6	14.4	9.43	9.12	38.8	85.4	W	D	5	WD	WD_5	WA
178	Hypnotod	F	688.6	27.5	9.818	9.487	37.9	83.4	W	D	5	WD	WD_5	MT
166	Jacob	M	349.6	14	9.906	9.636	25.1	55.2	W	D	5	WD	WD_5	WA
174	Lance	M	185.6	7.4	9.409	9.15	10.6	23.4	W	D	5	WD	WD_5	WA
169	Master Chief	M	286.6	11.5	9.534	9.272	20.5	45.1	W	D	5	WD	WD_5	WA
176	Nibbler	F	394.6	15.8	10.088	9.768	11.9	26.2	W	D	5	WD	WD_5	MT
179	Pandoodlek	F	463.6	18.5	9.837	9.536	47.4	104.2	W	D	5	WD	WD_5	MT
175	Robin	M	245.6	9.8	9.764	9.454	9.8	21.6	W	D	5	WD	WD_5	WA
170	Zoot	F	254.6	10.2	9.298	9.046	30.2	66.4	W	D	5	WD	WD_5	WA
82	Arthur	M	275.6	11	9.468	9.177	15.9	35	W	W	5	WW	WW_5	WA
78	Avery	M	213.6	8.5	9.629	9.318	22.9	50.4	W	W	5	WW	WW_5	WA
87	Bunn	M	346.6	13.9	9.267	9.003	7.2	15.9	W	W	5	WW	WW_5	MT
83	Caer	F	333.6	13.3	9.267	9.011	21.2	46.6	W	W	5	WW	WW_5	WA
77	Cortana	F	314.6	12.6	9.231	8.943	15.9	34.9	W	W	5	WW	WW_5	WA
90	David	M	405.6	16.2	9.228	8.942	15.5	34.1	W	W	5	WW	WW_5	MT
81	Herbert	M	360.6	14.4	9.517	9.149	11.1	24.5	W	W	5	WW	WW_5	WA
88	Hypnotod	F	688.6	27.5	9.638	9.08	38.7	85.1	W	W	5	WW	WW_5	MT
76	Jacob	M	349.6	14	9.424	9.115	24.3	53.4	W	W	5	WW	WW_5	WA
84	Lance	M	185.6	7.4	9.363	9.087	4	8.8	W	W	5	WW	WW_5	WA
79	Master Chief	M	286.6	11.5	9.848	9.429	9.6	21.2	W	W	5	WW	WW_5	WA
86	Nibbler	F	394.6	15.8	9.721	9.406	12.1	26.6	W	W	5	WW	WW_5	MT
89	Pandoodlek	F	463.6	18.5	9.278	9.901	52.1	114.7	W	W	5	WW	WW_5	MT
85	Robin	M	245.6	9.8	9.983	9.641	16.1	35.5	W	W	5	WW	WW_5	WA
80	Zoot	F	254.6	10.2	9.555	9.305	17.3	38.1	W	W	5	WW	WW_5	WA
367	Arthur	M	275.6	11	10.005	9.813	10.1	22.2	S	D	6	SD	SD_6	WA
363	Avery	M	213.6	8.5	9.389	9.262	17.1	37.6	S	D	6	SD	SD_6	WA
372	Bunn	M	346.6	13.9	9.798	9.671	1.5	3.3	S	D	6	SD	SD_6	MT
368	Caer	F	333.6	13.3	9.577	9.429	16.9	37.3	S	D	6	SD	SD_6	WA
362	Cortana	F	314.6	12.6	9.244	9.131	5.7	12.5	S	D	6	SD	SD_6	WA
375	David	M	405.6	16.2	9.314	9.203	14.8	32.5	S	D	6	SD	SD_6	MT
366	Herbert	M	360.6	14.4	9.548	9.411	18.3	40.3	S	D	6	SD	SD_6	WA

373	Hypnotod	F	688.6	27.5	9.072	8.969	18.5	40.7	S	D	6	SD	SD_6	MT
361	Jacob	M	349.6	14	9.583	9.465	11.8	26	S	D	6	SD	SD_6	WA
369	Lance	M	185.6	7.4	9.473	9.355	1.1	2.5	S	D	6	SD	SD_6	WA
364	Master Chief	M	286.6	11.5	9.298	9.183	9.6	21.2	S	D	6	SD	SD_6	WA
371	Nibbler	F	394.6	15.8	9.495	9.393	2.6	5.8	S	D	6	SD	SD_6	MT
374	Pandoodlek	F	463.6	18.5	9.533	9.426	41.3	90.9	S	D	6	SD	SD_6	MT
370	Robin	M	245.6	9.8	9.656	9.519	3.8	8.4	S	D	6	SD	SD_6	WA
365	Zoot	F	254.6	10.2	9.426	9.297	28.7	63.2	S	D	6	SD	SD_6	WA
277	Arthur	M	275.6	11	9.29	9.13	18.9	41.5	S	W	6	SW	SW_6	WA
273	Avery	M	213.6	8.5	9.719	9.554	27.9	61.3	S	W	6	SW	SW_6	WA
282	Bunn	M	346.6	13.9	9.699	9.539	10.1	22.3	S	W	6	SW	SW_6	MT
278	Caer	F	333.6	13.3	9.616	9.453	27.1	59.6	S	W	6	SW	SW_6	WA
272	Cortana	F	314.6	12.6	8.982	8.88	7.5	16.5	S	W	6	SW	SW_6	WA
285	David	M	405.6	16.2	9.568	9.414	26.1	57.5	S	W	6	SW	SW_6	MT
276	Herbert	M	360.6	14.4	9.373	9.224	13.4	29.4	S	W	6	SW	SW_6	WA
283	Hypnotod	F	688.6	27.5	9.481	9.358	19.9	43.8	S	W	6	SW	SW_6	MT
271	Jacob	M	349.6	14	9.797	9.653	12.2	26.9	S	W	6	SW	SW_6	WA
279	Lance	M	185.6	7.4	9.211	9.071	11.1	24.3	S	W	6	SW	SW_6	WA
274	Master Chief	M	286.6	11.5	9.295	9.142	17.8	39.1	S	W	6	SW	SW_6	WA
281	Nibbler	F	394.6	15.8	9.913	9.731	9.8	21.5	S	W	6	SW	SW_6	MT
284	Pandoodlek	F	463.6	18.5	9.903	9.737	53.6	117.9	S	W	6	SW	SW_6	MT
280	Robin	M	245.6	9.8	9.709	9.527	10.4	22.9	S	W	6	SW	SW_6	WA
275	Zoot	F	254.6	10.2	9.575	9.423	21.1	46.5	S	W	6	SW	SW_6	WA
187	Arthur	M	275.6	11	9.689	9.397	8.7	19.2	W	D	6	WD	WD_6	WA
183	Avery	M	213.6	8.5	9.417	9.15	37.5	82.4	W	D	6	WD	WD_6	WA
192	Bunn	M	346.6	13.9	9.715	9.421	1.5	3.3	W	D	6	WD	WD_6	MT
188	Caer	F	333.6	13.3	9.281	9.509	23.8	52.3	W	D	6	WD	WD_6	WA
182	Cortana	F	314.6	12.6	9.899	9.062	21.8	47.9	W	D	6	WD	WD_6	WA
195	David	M	405.6	16.2	9.952	9.68	5.8	12.7	W	D	6	WD	WD_6	MT
186	Herbert	M	360.6	14.4	8.989	8.749	23.3	51.3	W	D	6	WD	WD_6	WA
193	Hypnotod	F	688.6	27.5	9.488	9.21	42	92.3	W	D	6	WD	WD_6	MT
181	Jacob	M	349.6	14	9.154	8.912	23.1	50.9	W	D	6	WD	WD_6	WA
189	Lance	M	185.6	7.4	9.544	9.269	3.4	7.4	W	D	6	WD	WD_6	WA
184	Master Chief	M	286.6	11.5	9.288	9.03	15.2	33.4	W	D	6	WD	WD_6	WA
191	Nibbler	F	394.6	15.8	9.824	9.529	4.2	9.2	W	D	6	WD	WD_6	MT
194	Pandoodlek	F	463.6	18.5	9.797	9.507	30.4	66.8	W	D	6	WD	WD_6	MT
190	Robin	M	245.6	9.8	9.786	9.461	12.1	26.6	W	D	6	WD	WD_6	WA
185	Zoot	F	254.6	10.2	9.919	9.615	38.8	85.3	W	D	6	WD	WD_6	WA
97	Arthur	M	275.6	11	9.234	8.938	19.5	42.8	W	W	6	WW	WW_6	WA
93	Avery	M	213.6	8.5	8.946	8.707	16.1	35.4	W	W	6	WW	WW_6	WA
102	Bunn	M	346.6	13.9	9.507	9.22	15.3	33.6	W	W	6	WW	WW_6	MT
98	Caer	F	333.6	13.3	9.35	9.056	24.2	53.3	W	W	6	WW	WW_6	WA
92	Cortana	F	314.6	12.6	9.246	8.96	7.6	16.6	W	W	6	WW	WW_6	WA
105	David	M	405.6	16.2	9.555	9.266	24	52.8	W	W	6	WW	WW_6	MT

96	Herbert	M	360.6	14.4	9.245	8.94	20.5	45.1	W	W	6	WW	WW_6	WA
103	Hypnotod	F	688.6	27.5	9.867	9.428	22.9	50.4	W	W	6	WW	WW_6	MT
91	Jacob	M	349.6	14	9.369	9.081	12.4	27.3	W	W	6	WW	WW_6	WA
99	Lance	M	185.6	7.4	9.201	8.939	6.7	14.7	W	W	6	WW	WW_6	WA
94	Master Chief	M	286.6	11.5	9.009	8.773	14.6	32.1	W	W	6	WW	WW_6	WA
101	Nibbler	F	394.6	15.8	9.557	9.256	8.7	19	W	W	6	WW	WW_6	MT
104	Pandoodlek	F	463.6	18.5	9.27	8.986	35.3	77.6	W	W	6	WW	WW_6	MT
100	Robin	M	245.6	9.8	9.593	9.253	9.1	20	W	W	6	WW	WW_6	WA
95	Zoot	F	254.6	10.2	10.03	9.675	12	26.3	W	W	6	WW	WW_6	WA
7	Arthur	M	275.6	11	9.804	7.982	21.7	47.8	C	C	0	C	Start	WA
3	Avery	M	213.6	8.5	8.594	7.543	18.3	40.3	C	C	0	C	Start	WA
12	Bunn	M	346.6	13.9	11.425	9.366	7.6	16.6	C	C	0	C	Start	MT
8	Caer	F	333.6	13.3	12.158	9.499	24.9	54.9	C	C	0	C	Start	WA
2	Cortana	F	314.6	12.6	11.186	8.984	5.2	11.5	C	C	0	C	Start	WA
15	David	M	405.6	16.2	13.839	10.134	8.6	18.8	C	C	0	C	Start	MT
6	Herbert	M	360.6	14.4	12.526	9.324	50.2	110.4	C	C	0	C	Start	WA
13	Hypnotod	F	688.6	27.5	15.256	11.037	35.5	78.2	C	C	0	C	Start	MT
1	Jacob	M	349.6	14	12.206	9.519	23.1	50.8	C	C	0	C	Start	WA
9	Lance	M	185.6	7.4	9.714	8.56	5.3	11.6	C	C	0	C	Start	WA
4	Master Chief	M	286.6	11.5	11.72	9.477	6.7	14.6	C	C	0	C	Start	WA
11	Nibbler	F	394.6	15.8	11.197	9.51	4.2	9.3	C	C	0	C	Start	MT
14	Pandoodlek	F	463.6	18.5	12.696	9.654	27.4	60.2	C	C	0	C	Start	MT
10	Robin	M	245.6	9.8	8.689	7.764	22.1	48.6	C	C	0	C	Start	WA
5	Zoot	F	254.6	10.2	11.285	9.698	45	99	C	C	0	C	Start	WA
